# Annexin 1 Is a Component of eATP-Induced Cytosolic Calcium Elevation in *Arabidopsis thaliana* Roots

**DOI:** 10.3390/ijms22020494

**Published:** 2021-01-06

**Authors:** Amirah Mohammad-Sidik, Jian Sun, Ryoung Shin, Zhizhong Song, Youzheng Ning, Elsa Matthus, Katie A. Wilkins, Julia M. Davies

**Affiliations:** 1 Department of Plant Sciences, University of Cambridge, Cambridge CB2 3EA, UK; amirahbmsa@gmail.com (A.M.-S.); yn283@cam.ac.uk (Y.N.); ematthus@hotmail.com (E.M.); kaw67@cam.ac.uk (K.A.W.); 2 School of Life Sciences, Jiangsu Normal University, Xuzhou 221116, China; sunjian@jsnu.edu.cn; 3 RIKEN Centre for Sustainable Resource Science, Yokohama, Kanagawa 230-0045, Japan; ryoung.shin@riken.jp; 4 School of Agriculture, Ludong University, Yantai 264205, China; szhzh2000@163.com

**Keywords:** extracellular ATP, ADP, root, *Arabidopsis*, annexin 1, calcium, calcium channel, reactive oxygen species

## Abstract

Extracellular ATP (eATP) has long been established in animals as an important signalling molecule but this is less understood in plants. The identification of *Arabidopsis thaliana* DORN1 (Does Not Respond to Nucleotides) as the first plant eATP receptor has shown that it is fundamental to the elevation of cytosolic free Ca^2+^ ([Ca^2+^]_cyt_) as a possible second messenger. eATP causes other downstream responses such as increase in reactive oxygen species (ROS) and nitric oxide, plus changes in gene expression. The plasma membrane Ca^2+^ influx channels involved in eATP-induced [Ca^2+^]_cyt_ increase remain unknown at the genetic level. *Arabidopsis thaliana* Annexin 1 has been found to mediate ROS-activated Ca^2+^ influx in root epidermis, consistent with its operating as a transport pathway. In this study, the loss of function Annexin 1 mutant was found to have impaired [Ca^2+^]_cyt_ elevation in roots in response to eATP or eADP. Additionally, this annexin was implicated in modulating eATP-induced intracellular ROS accumulation in roots as well as expression of eATP-responsive genes.

## 1. Introduction

Extracellular ATP (eATP) is implicated as an apoplastic signal molecule in the abiotic and biotic stress responses of plants, their cellular viability, growth and stomatal regulation [1,2,3,4,5,6]. In *Arabidopsis thaliana*, eATP can act as a damage-associated molecular pattern (DAMP) and activates immunity signalling through the plasma membrane purinoreceptor AtDORN1 (Does Not Respond to Nucleotides1, also known as P2K1) [7]. A plasma membrane co-receptor P2K2 has recently been identified and both DORN1/P2K1 and P2K2 are lectin receptor kinases [8]. eATP-dependent but AtDORN1-independent effects have also been reported [9,10], pointing to the presence of other perception mechanisms. eATP perception triggers increase in root and leaf free cytosolic Ca^2+^ ([Ca^2+^]_cyt_) [5] that can lead to the production of reactive oxygen species (ROS) as further putative signalling agents [11,12,13,14]. Nitric oxide (NO) production can also be increased [2,15,16]. Eventually, signalling causes changes in gene expression [7,10,12,17,18,19]. Many eATP-responsive genes contain the CAM-box motif, which suggests that CAMTAs (Calmodulin-binding Transcription Activators) are important components [18]. Indeed, gene regulation could run through CAMTA3 [18], potentially connecting Ca^2+^ as a second messenger to changes in transcription due to eATP. It may be that the nuclear Ca^2+^ increase that follows eATP-induced [Ca^2+^]_cyt_ increase [20] activates CAMTA3 through Ca^2+^-CAM interaction. Understanding how eATP causes [Ca^2+^]_cyt_ increase is, therefore, relevant to downstream responses. 

It is known that eATP-induced [Ca^2+^]_cyt_ increase in *Arabidopsis* roots requires plasma membrane Ca^2+^ influx channels but the molecular identities of these channels remain unknown, as is also the case for other organs [9,11,14,21,22,23]. It has been hypothesized that the *Arabidopsis* annexin 1 protein (AtANN1) could be involved in mediating plasma membrane Ca^2+^ influx [5,24] and recently AtANN4 was found to mediate eATP-induced [Ca^2+^]_cyt_ increase when expressed in *Xenopus* oocytes [25]. AtANN1 is thought to act as a plasma membrane Ca^2+^ channel in the root [Ca^2+^]_cyt_ response to salinity stress, hyperosmotic stress, and oxidative stress [26,27,28]. In this study, the possible involvement of AtANN1 in the root and leaf eATP-[Ca^2+^]_cyt_ signalling pathway has been tested using an *Atann1* loss of function mutant constitutively expressing cytosolic (apo)aequorin as a luminescent [Ca^2+^]_cyt_ reporter [26,27,28]. As eADP has also been shown to increase [Ca^2+^]_cyt_ [7,11,12,14,21], this nucleotide was also tested. The consequences for eATP-induced root intracellular ROS elevation and gene transcription were also investigated. The results show that AtANN1 is required for the normal [Ca^2+^]_cyt_ response towards both eATP and eADP in roots. It affects the spatial extent of intracellular ROS accumulation in roots and influences their eATP-induced transcriptional response.

## 2. Results

### 2.1. AtANN1 Mediates Root [Ca^2+^]_cyt_ Elevation In Response To eATP and eADP

**Figure 1:**
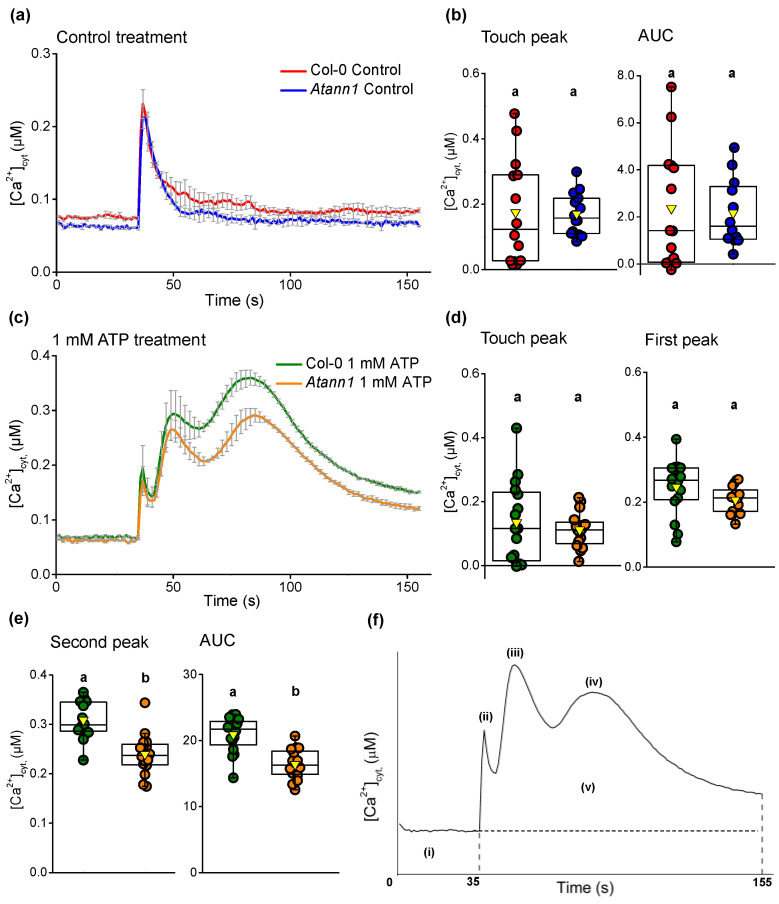
*Arabidopsis* annexin 1 (AtANN1) is needed for normal [Ca^2+^]_cyt_ elevation in a root by eATP. (**a**) Time course of [Ca^2+^]_cyt_ elevation produced by control treatment in three experiments (mean (± SEM): Col-0 in red, *n* = 14 roots in total; *Atann1* loss of function mutant in blue, *n* = 14). (**b**) The [Ca^2+^]_cyt_ touch peak values and area under the curve (AUC) extracted from the control time course (± SEM). Middle line of the boxplot represents the median whereas the inverted triangle represents the mean. (**c**) Time course of [Ca^2+^]_cyt_ elevation with 1 mM eATP treatment in 3 experiments (Col-0 in green, *n* = 16; *Atann1* in orange, *n* = 16). (**d**) The touch peak and the first peak [Ca^2+^]_cyt_ values extracted from the 1 mM eATP time course. (**e**) Second peak and the AUC [Ca^2+^]_cyt_ values. (**f**) Schematic diagram of different time course sections. Each section was calculated with the average baseline value (indicated by (i)) subtracted. Touch peak (ii) was the highest [Ca^2+^]_cyt_ value of the touch response between 35 and 40 s due to mechanical stimulus from solution addition at the 35th second. First peak (iii) and second peak (iv) were the highest [Ca^2+^]_cyt_ value between 40 s and 60 s and 60 s and 155 s, respectively. Total [Ca^2+^]_cyt_ accumulation was obtained from the AUC (v; 35 s–155 s). *p*-values were obtained from analysis of variance (ANOVA) with Tukey’s post-hoc test or Kruskal–Wallis test for non-parametric approaches. Different lower-case letters indicate a significant difference between means (*p* < 0.05).

Previously, eATP-dependent root [Ca^2+^]_cyt_ elevation was found to be wholly reliant on the AtDORN1 receptor [5]. To assess the role of AtANN1 in this response, seven-day-old whole roots of *Atann1* (loss of function mutant) and Col-0 (expressing cytosolic (apo)aequorin under the 35S CaMV promoter) were excised and assayed individually to measure [Ca^2+^]_cyt_ in the presence of eATP. Addition of control solution after 35 s of measurement evoked a monophasic [Ca^2+^]_cyt_ increase in response to mechanical stimulation (“touch response”) before returning to the basal level (Figure 1a). The touch response of *Atann1* roots was similar to Col-0 in terms of the amplitude (“touch peak”) and the total accumulation of [Ca^2+^]_cyt_ (estimated as the area under the curve, AUC) (*p* > 0.05; Figure 1b). Measurement of [Ca^2+^]_cyt_ in response to 1 mM eATP revealed a biphasic increase comprising a first peak and second peak after the initial touch response (Figure 1c). A biphasic response in roots was also observed previously [5,11]. The touch response was similar between genotypes (*p* > 0.5) and although the first eATP peak was lower in *Atann1* it was not significantly different to Col-0 (*p* > 0.05) (Figure 1d). The second eATP-induced [Ca^2+^]_cyt_ peak response of *Atann1* was significantly lower than Col-0 (*p* < 0.0001) (Figure 1e). The total [Ca^2+^]_cyt_ accumulated was also significantly lower in *Atann1* (*p* < 0.0001; Figure 1e).

**Figure 2:**
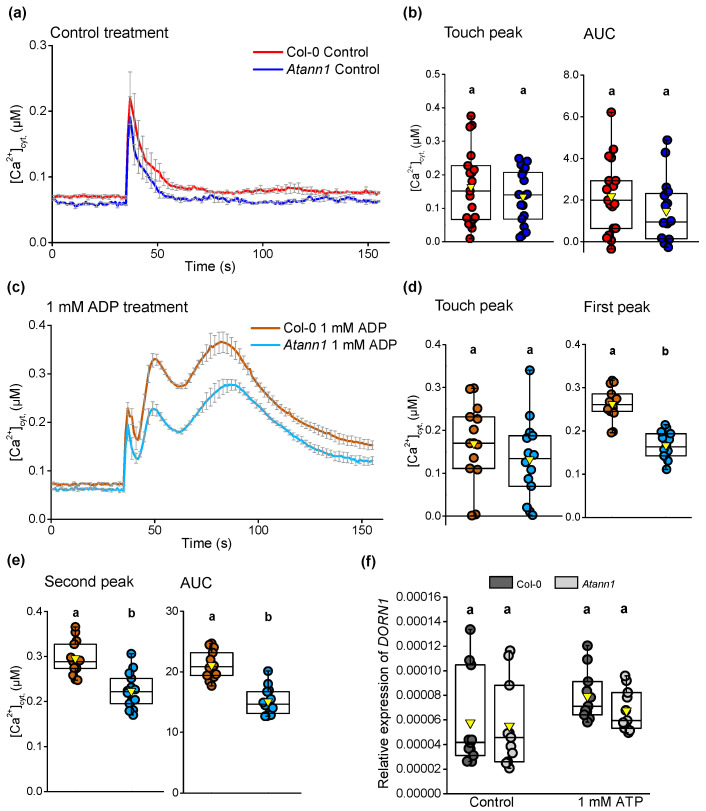
AtANN1 is involved in eADP-induced [Ca^2+^]_cyt_ elevation in the root. (**a**) Mean (± SEM) time course of [Ca^2+^]_cyt_ increase by control treatment in three experiments (Col-0 in red, *n* = 19; *Atann1* in blue, *n* = 18). (**b**) [Ca^2+^]_cyt_ touch peak values and AUC extracted from the control time course. Middle line of the boxplot represents the median whereas the inverted triangle represents the mean. (**c**) Mean (± SEM) time course of [Ca^2+^]_cyt_ increase by 1 mM eADP treatment obtained from three experiments (Col-0 in brown, *n* = 14; *Atann1* in light blue, *n* = 14). (**d**) The touch peak and the first peak [Ca^2+^]_cyt_ values from the 1 mM eADP time course. (**e**) Second peak and the total AUC [Ca^2+^]_cyt_ values. (**f**) Quantification of *AtDORN1* gene expression in Col-0 and *Atann1* roots after seven days of growth on control medium or 1 mM eATP-containing medium (Col-0 in black, *Atann1* in grey with *n* = 11 for each genotype and treatment) obtained from three experiments. *p*-values were obtained from ANOVA with Tukey’s post-hoc test. Different lower-case letters indicate a significant difference between means (*p* < 0.05).

eADP also evokes a biphasic [Ca^2+^]_cyt_ increase in roots [5,11] that is entirely dependent on AtDORN1 [5]. To assess whether AtANN1 is also required, a similar test with eADP on seven-day-old excised roots was carried out for Col-0 and *Atann1.* As shown in Figure 2a, control treatment elicited a monophasic touch response in both genotypes. No significant differences were evident for either the touch peak (*p* > 0.05) or the overall [Ca^2+^]_cyt_ (*p* > 0.05) between Col-0 and *Atann1* (Figure 2b). In the presence of 1 mM eADP as shown in Figure 2c, both Col-0 and *Atann1* produced a biphasic [Ca^2+^]_cyt_ increase following the touch response. No significant difference was found between Col-0 and *Atann1* in the touch peak response (*p* > 0.05) (Figure 2d). Unlike eATP treatment however, *Atann1* showed a significantly impaired ability to produce both a normal first peak in response to 1 mM eADP (*p* < 0.0001) (Figure 2d) and a normal second peak (*p* < 0.0001) (Figure 2e). Overall, the loss of functional AtANN1 protein led to a reduced total accumulation of [Ca^2+^]_cyt_ compared to Col-0 (*p* < 0.0001) (Figure 2e). Lowered *AtDORN1* expression cannot explain the impairments in *Atann1′s* response to extracellular nucleotides as no significant difference in the receptor’s expression between Col-0 and *Atann1* roots was found in either control conditions (*p* > 0.5) or in the presence of 1 mM ATP (*p* > 0.5) (Figure 2f). Therefore, the defects in [Ca^2+^]_cyt_ elevation appear to rest with the lack of AtANN1.

### 2.2. AtANN1’s Involvement in the eATP-Generated First Peak Response Is Concentration-Dependent

**Figure 3:**
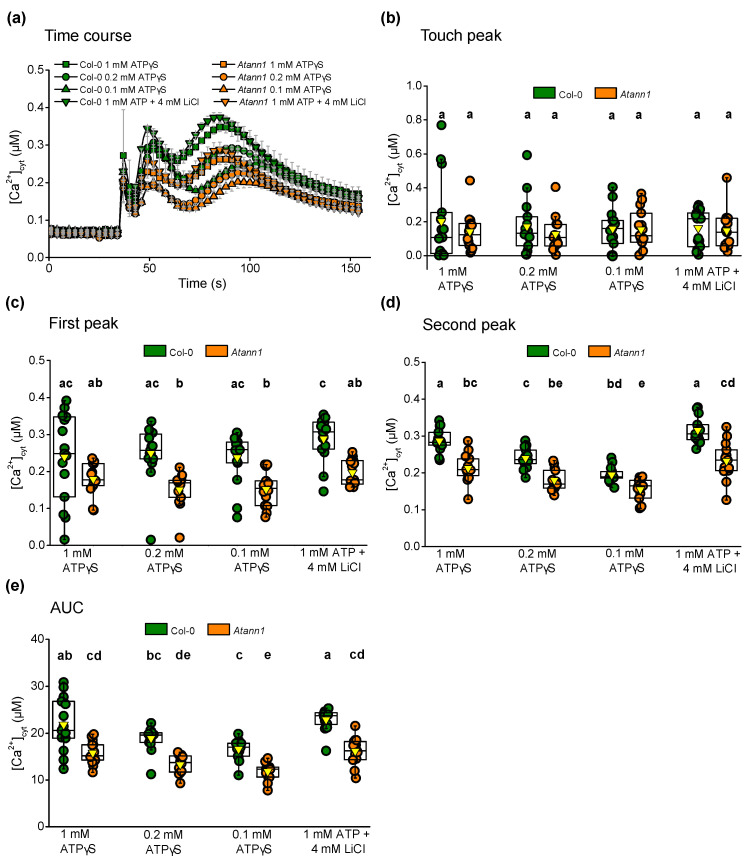
AtANN1 is crucial for the first peak [Ca^2+^]_cyt_ response at lower eATP concentration. (**a**) Mean (± SEM) [Ca^2+^]_cyt_ time course in response to different concentrations of eATPγS or eATP with LiCl control from 3 experiments with *n* = 14–15 per genotype and treatment. (**b**) The [Ca^2+^]_cyt_ touch peak values (± SEM), (**c**) first peak [Ca^2+^]_cyt_ values (± SEM), (**d**) second peak [Ca^2+^]_cyt_ values (± SEM) and (**e**) the total [Ca^2+^]_cyt_ accumulated obtained from AUC (± SEM) for each concentration tested in both Col-0 and *Atann1* extracted from the time course. *p*-values were obtained from ANOVA with Tukey’s post-hoc test or Kruskal-Wallis test for non-parametric approach. Different lower case letters indicate significant difference between means (*p* < 0.05).

A non-hydrolysable ATP analogue (ATPγS; Adenosine 5′-[γ-thio] triphosphate) and ADP analogue (ADPβS; Adenosine 5′-[β-thio] diphosphate) were then used to confirm that the agonists acted as signal molecules rather than as energy sources that drive the [Ca^2+^]_cyt_ increase. The ATPγS used was a tetralithium salt whereas the ADPβS was a trilithium salt. A LiCl treatment (4 mM for ATPγS and 3 mM for ADPβS) was carried out alongside the non-hydrolysable analogues as a lithium control. Seven-day-old individual whole roots responded with the biphasic [Ca^2+^]_cyt_ increase when tested with different concentrations of eATPγS, or the eATP/LiCl salt control (Figure 3a). As seen in Figure 3b, the touch peak responses were not significantly different between genotypes (*p* > 0.5). The Col-0 first [Ca^2+^]_cyt_ peak did not require eATP hydrolysis (no significant difference between 1 mM eATPγS and 1 mM eATP/LiCl) and indeed was already “saturated” at 0.1 mM eATPγS (no significant difference, *p* > 0.05, between 0.1 and 1 mM eATPγS) (Figure 3c). The first peak responses of *Atann1* also appeared saturated at 0.1 mM eATPγS (Figure 3c). As in the response to 1 mM eATP in Figure 1d, the first peak of *Atann1* in response to 1 mM eATPγS was lower than Col-0 but was not significant (Figure 3c). However, when 1 mM eATP was tested with LiCl as a control for Li^+^ addition, the difference did become significant. This may be due to the range of Col-0 values in the 1 mM eATPγS test. Importantly, within each genotype there was no evidence for eATP’s acting as an energy source at 1 mM. *Atann1* showed a significantly lower first peak [Ca^2+^]_cyt_ response compared to Col-0 in response to 0.1 mM eATPγS (*p* < 0.001) and 0.2 mM eATPγS (*p* < 0.001) (Figure 3c). These results suggest that the role of AtANN1 in the first peak response relies on the concentration of agonist used. The second [Ca^2+^]_cyt_ peak of Col-0 showed a significant dependence on the concentration of eATPγS, as did *Atann1* between 0.1 and 1 mM (Figure 3d). AtANN1 proved to be important in generating the second peak as the *Atann1* mutant failed to respond in similar magnitude as the Col-0 over the concentration range (*p* < 0.001) (Figure 3d). This was also evident in the total [Ca^2+^]_cyt_ accumulated where there were significant differences between Col-0 and *Atann1* (*p* < 0.0001) in the AUC for every concentration tested (Figure 3e).

**Figure 4:**
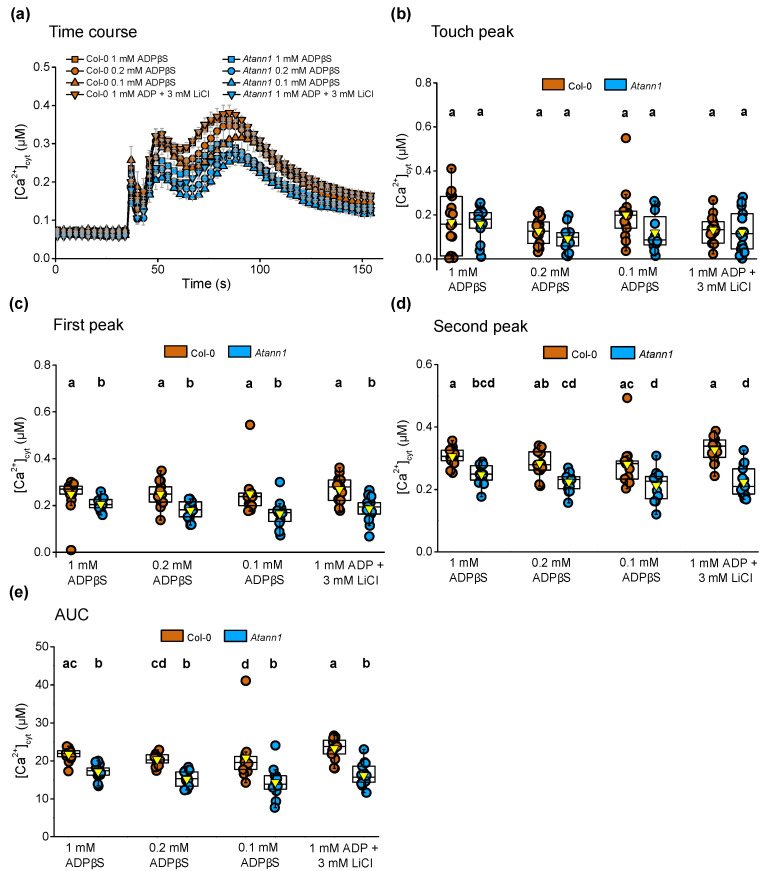
AtANN1 is involved in the eADPβS-induced [Ca^2+^]_cyt_ response at all concentrations tested. (**a**) Mean (± SEM) [Ca^2+^]_cyt_ time course in response to different concentrations of eADPβS or ADP with LiCl control from 3 experiments (*n* = 13—15 per genotype and treatment). (**b**) The [Ca^2+^]_cyt_ touch peak values (± SEM), (**c**) first peak [Ca^2+^]_cyt_ values (± SEM), (**d**) second peak [Ca^2+^]_cyt_ values (± SEM) and (**e**) the total [Ca^2+^]_cyt_ accumulated obtained from AUC (± SEM) for each concentration tested in both Col-0 and *Atann1* extracted from the time courses. *p*-values were obtained from ANOVA with Tukey’s post-hoc test or Kruskal-Wallis test for non-parametric approach. Different lower case letters indicate a significant difference between means (*p* < 0.05).

Both Col-0 and *Atann1* generated the transient biphasic [Ca^2+^]_cyt_ elevation after the touch peak response when challenged with different concentrations of eADPβS or 1 mM eADP with 3 mM LiCl (Figure 4a). Both Col-0 and *Atann1* produced the same level of touch peak response for each treatment (*p* > 0.5) (Figure 4b). Just like the first peak response to eATPγS, the response of both genotypes to eADPβS appeared saturated at 0.1 mM (Figure 4c). In contrast to the eATPyS test, there were significant differences between Col-0 and *Atann1* in the first peak [Ca^2+^]_cyt_ response regardless of the concentration of eADPβS tested (*p* < 0.001) (Figure 4c). Consistent with the results in the hydrolysable eADP test, *Atann1* was found to produce lower [Ca^2+^]_cyt_ responses than the Col-0 in the second peak (*p* < 0.001) (Figure 4d) and in the AUC (*p* < 0.001) (Figure 4e) for all the concentrations tested. There was no evidence for eADP’s acting as an energy source. Overall, these results suggest that the involvement of AtANN1 in generating the first peak response is specific to lower concentrations of eATP, with the likelihood of other components participating at higher concentration. AtANN1 is still needed for the first peak response to high concentration of eADP and for the second peak regardless of agonist concentration.

### 2.3. AtANN1 Sets the Spatial Extent of Intracellular ROS in Roots in Response to eATP

**Figure 5:**
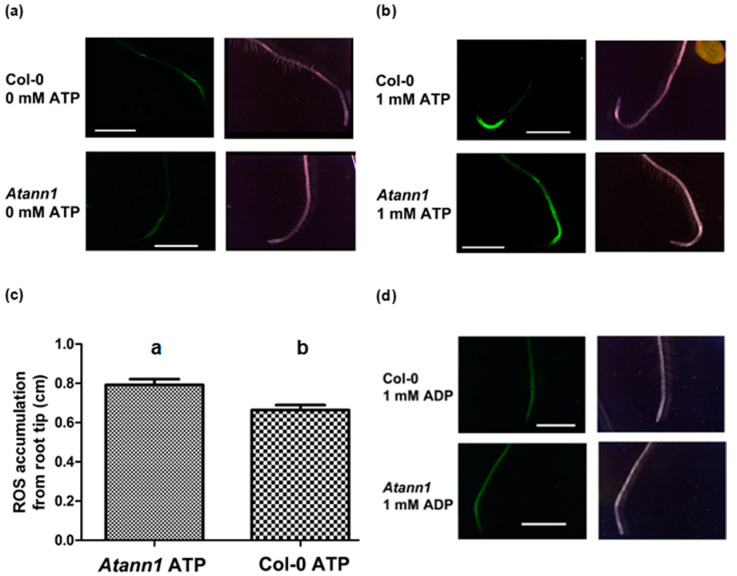
*Atann1* supports a longer zone of intracellular ROS accumulation than Col-0 in response to eATP. (**a**) CM-H2DCFDA fluorescence from a Col-0 or *Atann1* root under control conditions. Corresponding bright field images are also shown. (**b**) Roots after exposure to 1 mM eATP. (**c**) Mean (± SEM) length of root from the tip fluorescing after exposure to eATP (Col-0 *n* = 48; *Atann1 n* = 68; *p* = 0.0026, Student’s *t*-test). (**d**) Roots after exposure to 1 mM eADP. Scale bar = 4 mm. Different lower-case letters indicate a significant difference between means (*p* < 0.05).

Addition of eATP (but not eADP) causes rapid intracellular accumulation of ROS in *Arabidopsis* roots that requires Ca^2+^ influx and is largely dependent on AtRBOHC activity [14,21]. Whether AtANN1 is involved in the production of intracellular ROS was tested here with ester-loaded CM-H_2_DCFDA (5-(and-6-)-chloromethyl-2′,7′-dichlorodihydrofluorscein diacetate) [14]. Figure 5a shows the baseline ROS detected in control conditions. In the presence of 1 mM eATP (Figure 5b), ROS increase was detectable within 20 s, as reported previously [14]. Signal intensity was higher than the baseline in both Col-0 and *Atann1* with the latter supporting a greater length of ROS production that clearly extended into the mature zone supporting root hairs (Figure 5b). Further statistical analysis carried out confirmed this significant difference between *Atann1* and Col-0 (Figure 5c). In line with previous studies [14,21], 1 mM eADP failed to induce any intracellular ROS accumulation in either genotype (Figure 5d).

**Figure 6:**
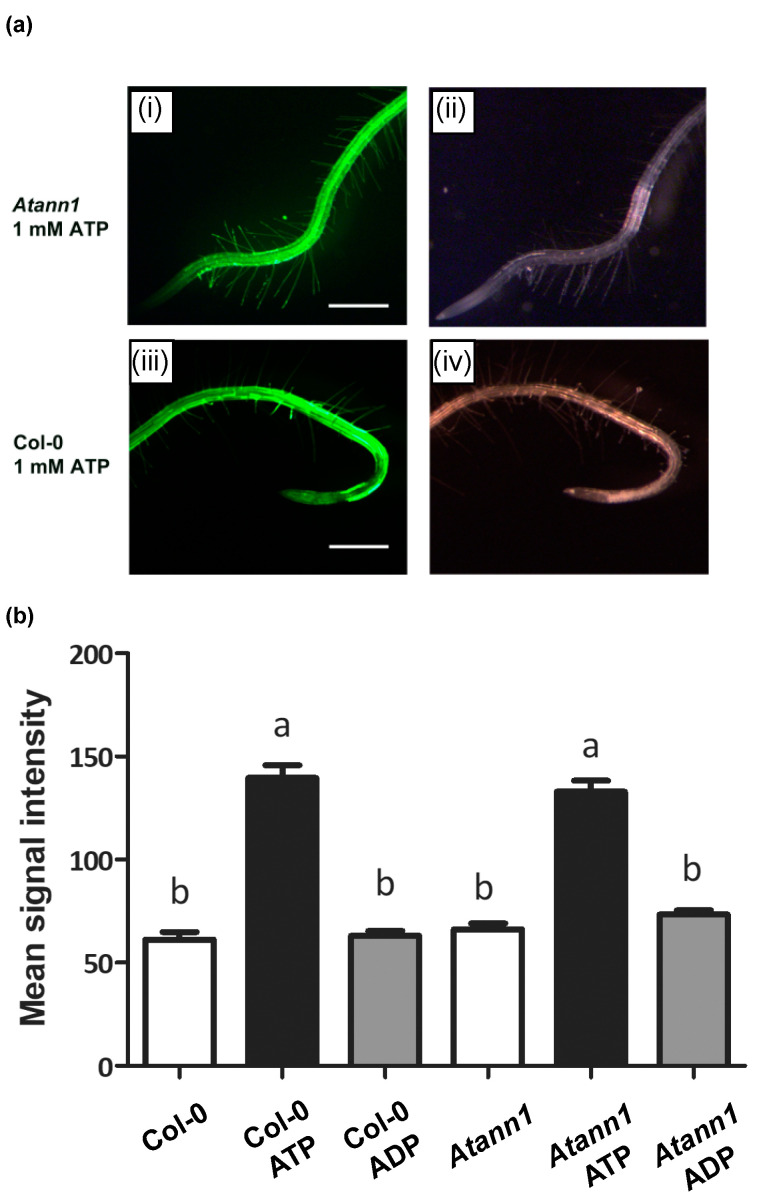
eATP-induced intracellular ROS accumulation at the root apex at higher resolution. (**a**) CM-H_2_DCFDA fluorescence from (i) a representative *Atann1* root exposed to 1 mM eATP and (ii) corresponding bright field image. (iii) A representative Col-0 root after exposure to 1 mM eATP and (iv) corresponding bright field image. (**b**) Mean (± SEM) of fluorescence pixel intensity at root apices under control conditions and after 30 s exposure to 1 mM eATP (Col-0 *n* = 48; *Atann1 n* = 68) or 1 mM ADP (Col-0 *n* = 32; *Atann1 n* = 38). Both genotypes responded significantly to eATP but not eADP (*p* < 0.001; ANOVA with Dunnett’s post-hoc test). Scale bar = 2 mm. Different lower-case letters indicate a significant difference between means (*p* < 0.05).

The focus of the analysis was then shifted to the root apex to distinguish any differences in signal intensity between Col-0 and *Atann1* (Figure 6a). Based on the mean signal intensity, there was no significant difference in ROS production between genotypes under control conditions. Both Col-0 and *Atann1* treated with 1 mM eATP produced significantly higher ROS than under control conditions but although *Atann1* supported a greater spatial extent of ROS accumulation, the mean signal intensity at the root apex was similar to Col-0 in the presence of eATP. Once again, 1 mM eADP treatment failed to increase ROS in either genotype (Figure 6b). Overall, these data suggest that AtANN1 is involved in controlling the spatial extent of ROS accumulation evoked by 1 mM eATP.

### 2.4. AtANN1 Is Required For eATP-Induced Changes in the Expression of ACS6 and WRKY40

**Figure 7:**
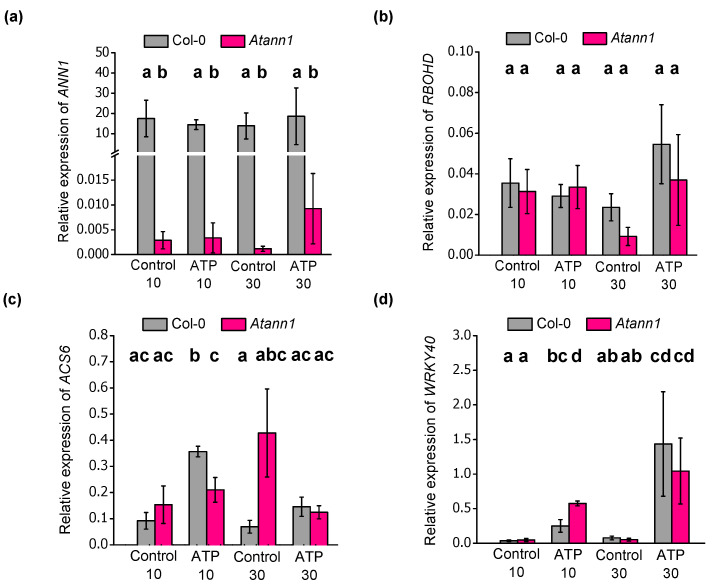
Transcriptional regulation of stress responsive genes by AtANN1. Col-0 and *Atann1* whole roots were treated with control solution or 1 mM eATP for 10 or 30 min. Transcript abundance of (**a**) *AtANN1*, (**b**) *AtRBOHD*, (**c**) *AtACS6* and (**d**) *AtWRKY40* normalised to two housekeeping genes; *AtUBQ10* and *AtTUB4*. Data were from the means (± SEM) of four independent trials. Student’s *t*-test and Welch’s *t*-test were used to test parametric data whereas Wilcoxon rank sum test was used for non-parametric data. Different lower-case letters indicate a significant difference between means (*p* < 0.05).

eATP has been shown previously to be able to induce transcription of genes involved in stress responses [7,10,12,18,29,30,31]. eATP-induced genes *AtRBOHD* (NADPH/Respiratory Burst Oxidase Protein D), *AtWRKY40* (WRKY DNA-Binding Protein 40) and *AtACS6* (1-Aminocyclopropane-1-carboxylic Acid Synthase 6) [7,12,18] were tested for regulation by 1 mM eATP in roots and the possibility of AtANN1′s affecting their regulation (Figure 7). Ionic composition of the control solution was identical to that used in the aequorin tests (10 mM CaCl_2_, 0.1 mM KCl). *AtANN1* transcript was almost completely knocked-down in *Atann1* compared to the Col-0 in control conditions (*p* < 0.05) and after eATP treatment (*p* < 0.01). Transcript level did not increase in Col-0 after eATP treatment (Figure 7a). *AtRBOHD* gene was not up-regulated by either 10 min or 30 min of eATP treatment when compared with the control for both Col-0 (*p* > 0.05) and *Atann1* (*p* > 0.05) (Figure 7b). In contrast, *AtACS6* was significantly upregulated in Col-0 when treated for 10 min with eATP compared to the control treatment (*p* < 0.01) but fell back to control levels after 30 min (*p* > 0.05; Figure 7c). Expression was not significantly upregulated in *Atann1* after 10 min of eATP treatment (*p >* 0.5) and it remained significantly lower than Col-0 at this time point (*p* < 0.05). No significant difference was evident in *AtACS6* expression between 30 min control treatment and eATP treatment for *Atann1* (*p* > 0.05; Figure 7c). These findings suggest a temporal regulation of *AtACS6* by AtANN1 in the presence of eATP.

Transcript abundance of *AtWRKY40* was upregulated by eATP exposure (Figure 7d). Col-0 and *Atann1* samples treated with eATP for 10 and 30 min had significantly higher *AtWRKY40* transcript abundance compared to their controls (Col-0, *p* < 0.05; *Atann1*, *p* < 0.05). Notably, *Atann1* supported a significantly higher increase in *AtWRKY40* transcript than Col-0 after 10 min of eATP treatment (*p* < 0.05) but over time, no significant difference in transcript abundance between genotypes was found after 30 min of eATP treatment (*p* > 0.5) (Figure 7c), suggesting a temporal effect on the response. Overall, AtANN1 appears important in regulating the eATP-induced changes in the transcription of *AtACS6* and *AtWRKY40.*

### 2.5. eATP-Induced [Ca^2+^]_cyt_ Elevation Is Not Mediated by AtANN1 in Cotyledons

**Figure 8:**
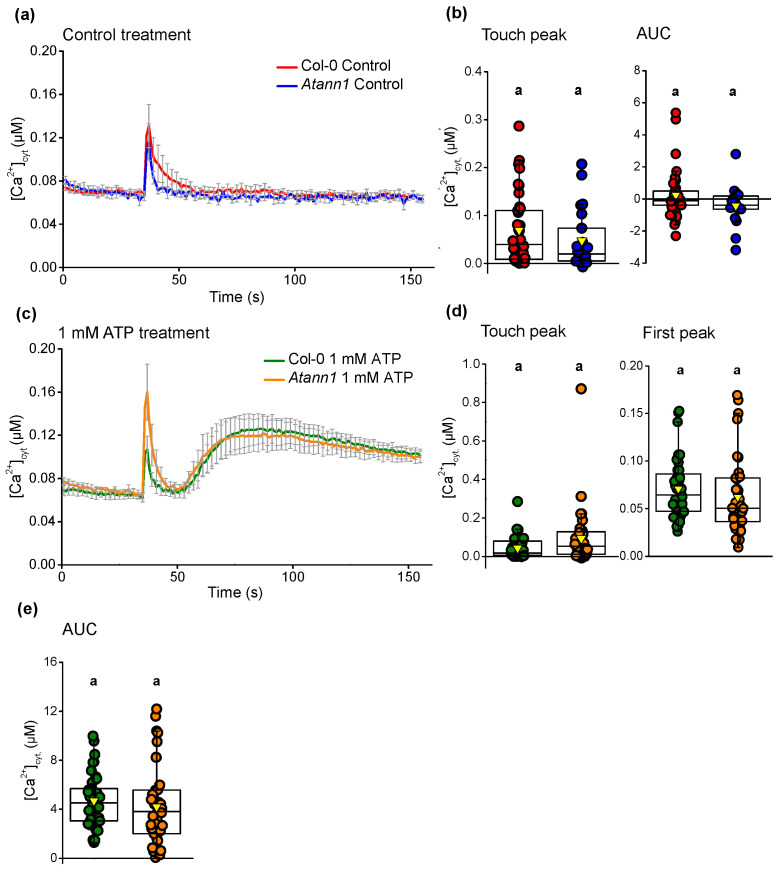
AtANN1 is not needed for eATP-induced [Ca^2+^]_cyt_ increase in cotyledons. (**a**) Mean (± SEM) time course of [Ca^2+^]_cyt_ increase due to control treatment of seven-day-old individual cotyledons. (**b**) The [Ca^2+^]_cyt_ touch peak (± SEM) and the total [Ca^2+^]_cyt_ accumulation by the AUC (± SEM). Middle line of the boxplot represents the median whereas the inverted triangle represents the mean. (**c**) Mean (± SEM) time course of [Ca^2+^]_cyt_ increase due to 1 mM eATP treatment. (**d**) The [Ca^2+^]_cyt_ touch peak values (± SEM) and the first peak values (± SEM). (**e**) Total [Ca^2+^]_cyt_ accumulated values (± SEM) from the AUC. Data were obtained from four experiments with *n* = 21–39 cotyledons per genotype and treatment. *p*-values obtained from ANOVA with Tukey’s post-hoc test. Identical lower-case letters indicate an insignificant difference between means (*p* > 0.05).

Whole seedlings of *A. thaliana* were found previously to elevate [Ca^2+^]_cyt_ in response to eATP [7]. To assess whether AtANN1 is involved in mediating this response in aerial organs as well as roots, the [Ca^2+^]_cyt_ elevation by 1 mM eATP of seven-day-old cotyledons of Col-0 and *Atann1* was compared. Consistent with previous results on Col-0 true leaves [5], control treatment of cotyledons caused a monophasic touch response in both Col-0 and *Atann1* (Figure 8a) that was not significantly different between genotypes (*p* > 0.05; Figure 8b). Also in common with previous results on Col-0 true leaves [5], 1 mM eATP treatment of cotyledons caused a prolonged monophasic [Ca^2+^]_cyt_ increase after the touch response (Figure 8c). No significant differences were found between Col-0 and *Atann1* in either the touch peak (*p* > 0.05), the first peak (*p* > 0.05) or in the total [Ca^2+^]_cyt_ accumulated (*p* > 0.05; Figure 8d,e). Based on these observations, it is certain that AtANN1′s role in eATP signalling does not extend to the cotyledon and that the [Ca^2+^]_cyt_ signature caused by eATP treatment differs with the type of tissues or organs tested.

### 2.6. AtANN1 Is Less Important in the [Ca^2+^]_cyt_ Response of True Leaves to Extracellular ATP or ADP

**Figure 9:**
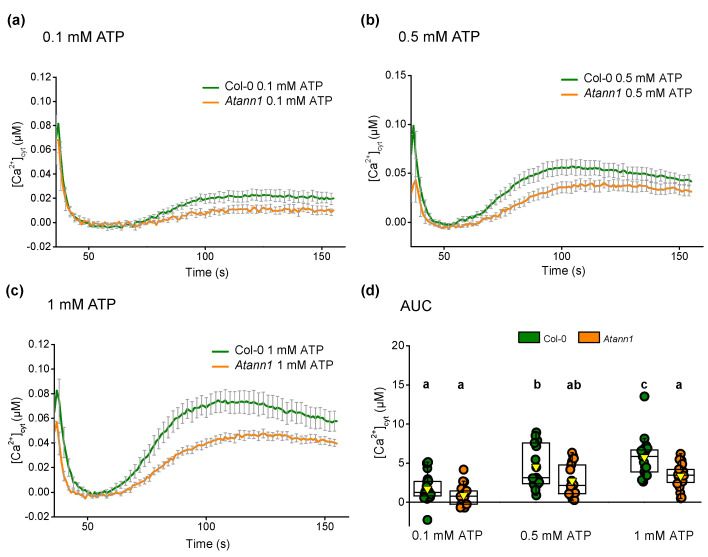
AtANN1 is required for leaf [Ca^2+^]_cyt_ elevation at higher concentrations of eATP. Mean (± SEM) [Ca^2+^]_cyt_ time course (t = 36 s–155 s) of 14-day-old individual true leaves treated with (**a**) 0.1 mM eATP, (**b**) 0.5 mM eATP or (**c**) 1 mM eATP. (**d**) The total [Ca^2+^]_cyt_ accumulated over the period of measurement for each concentration given as AUC. Data were obtained from four experiments with *n* = 17–22 per genotype and treatment. Middle line of the boxplot represents the median whereas the inverted triangle represents the mean. *p*-values were obtained from ANOVA with Tukey’s post-hoc test. Different lower-case letters indicate a significant difference between means (*p* < 0.05).

*AtANN1* expression is evident in true leaves as well as cotyledons [32,33,34]. To assess whether AtANN1′s participation in [Ca^2+^]_cyt_ elevation is limited to roots, individual 14-days-old leaves were tested with varying concentrations of eATP and eADP. All the eATP concentrations used (0.1 mM, Figure 9a; 0.5 mM, Figure 9b; 1 mM, Figure 9c) generated a prolonged monophasic [Ca^2+^]_cyt_ response after the touch peak that was similar to both the seven-day-old cotyledon [Ca^2+^]_cyt_ pattern in the previous test and 14-day-old Col-0 leaves studied previously [5]. Although *Atann1* produced a lower peak response than Col-0, differences between the genotypes were not significant except for 1 mM eATP’s causing Col-0 to produce a significantly higher total [Ca^2+^]_cyt_ than *Atann1* (*p* < 0.05) (Figure 9d).

**Figure 10:**
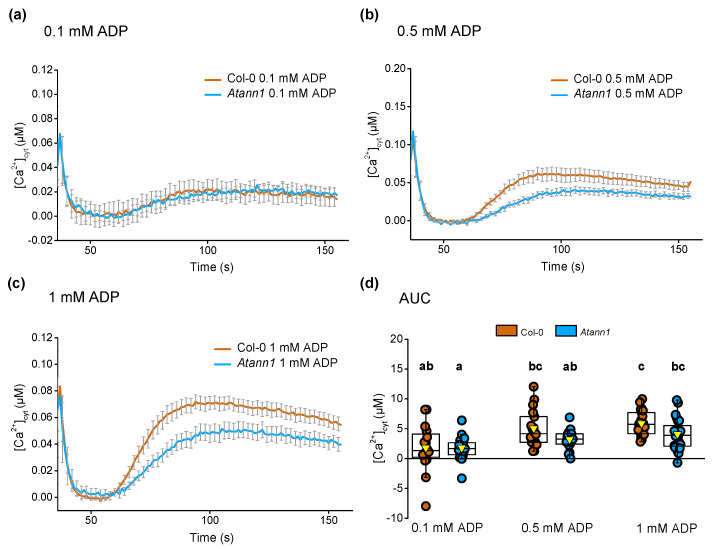
AtANN1 is not involved in mediating eADP-induced [Ca^2+^]_cyt_ increase in leaves. Mean (± SEM) time course of [Ca^2+^]_cyt_ increase (t = 36s–155s) when 14-day-old individual leaves were treated with (**a**) 0.1 mM eADP, (**b**) 0.5 mM eADP or (**c**) 1 mM eADP agonist. (**d**) The total [Ca^2+^]_cyt_ accumulated over the period of measurement for each concentration by calculating the AUC. Data were obtained from four experiments (Col-0 *n* = 16–23; *Atann1 n* = 17–24). The middle line of the boxplot represents the median whereas the inverted triangle represents the mean. *p*-values were obtained from ANOVA with Tukey’s post-hoc test. Different lower-case letters indicate a significant difference between means (*p* < 0.05).

A similar pattern was found in tests of eADP, which evoked a monophasic [Ca^2+^]_cyt_ response at 0.1 mM (Figure 10a), 0.5 mM (Figure 10b) and 1 mM ADP (Figure 10c). Although *Atann1* leaf samples had lower peak [Ca^2+^]_cyt_ responses than Col-0, these were not significantly different and no significant differences were found between Col-0 and *Atann1* for the total [Ca^2+^]_cyt_ accumulated at each concentration (in all cases *p* > 0.05) (Figure 10d). Thus, at this level of resolution, AtANN1 appears only to have an impact in leaves at 1 mM eATP.

## 3. Discussion

Few components of eATP (or eADP) signalling pathways have been identified at the genetic level. In this study, the evidence suggests that AtANN1 is a component of both eATP- and eADP-induced [Ca^2+^]_cyt_ elevation in roots, with consequences for eATP-induced ROS accumulation and gene expression. Mechanical or “touch” stimulus can cause accumulation of extracellular ATP by *Arabidopsis* root tips but much less so in older regions of the root [35]. Here, the addition of control solution alone caused a touch-induced monophasic increase in [Ca^2+^]_cyt_ (Figure 1a) but it is not known whether it also caused accumulation of extracellular ATP to trigger all or part of that [Ca^2+^]_cyt_ increase. There was no significant difference between the touch response of Col-0 and *Atann1,* which may indicate that AtANN1 does not contribute to any [Ca^2+^]_cyt_ increase that is downstream of any touch-induced eATP increase. There were no indications of further [Ca^2+^]_cyt_ elevations in response to addition of control solution, suggesting that any extracellular ATP produced by that mechanical stimulus was insufficient to trigger the biphasic [Ca^2+^]_cyt_ increase seen when ATP is added experimentally. However, it cannot be ruled out that it could affect the root’s subsequent response to experimental addition of nucleotides. Previous analysis of the Col-0 root’s biphasic [Ca^2+^]_cyt_ response to experimental additions of eATP or eADP demonstrated that this biphasic pattern is wholly reliant on AtDORN1 as an eATP receptor [5]. For eATP, the first peak originates at the root apex whereas the second peak originates sub-apically in more mature cells, possibly as part of a [Ca^2+^]_cyt_ “wave” that travels from the apex [5,20,36,37,38]. Mature regions can also respond to eATP when it is added there specifically rather than to the whole root [5], showing a level of autonomy from the apex. It is assumed that the biphasic eADP response maps to the same areas. AtANN1 is present at the root apex and in more mature cells such as trichoblasts [32,34] so it could contribute to both phases. The near normal first peak [Ca^2+^]_cyt_ in the *Atann1* mutant with 1 mM eATP (Figure 1d, Figure 3c) but not with 0.2 mM or 0.1 mM eATPγS (Figure 3c) suggests that at high concentration, other components can compensate for the loss of AtANN1 especially in the root apex. No such redundancy was observed with eADP. A far clearer need for AtANN1 was seen in the second eATP- and eADP-induced [Ca^2+^]_cyt_ increase across the concentration range tested and this could map to mature cells. A study of *Atann1* using a [Ca^2+^]_cyt_ reporter affording spatial resolution such as YC3.6 (Yellow Cameleon 3.6) or root cell-specific GCaMP3 [20,36,37,38,39] is now needed to determine which cells and regions AtANN1 operates in. Challenging specific regions of the root with agonist could also help determine whether an impaired apical/first peak response leads to an impaired sub-apical/second peak response (which could help explain the patterns observed here) and the extent to which the lesion in the *Atann1* second peak is a consequence of a local response to agonist independent of the apex.

In *Arabidopsis* roots, eATP (but not eADP) causes intracellular ROS accumulation, which relies on Ca^2+^ influx and the AtRBOHC NADPH oxidase [14,21]. Another source of ROS production is AtRBOHD, which is a phosphorylation target of AtDORN1 in guard cells [7,40]. It is envisaged that Ca^2+^ influx acts downstream of AtDORN1 and upstream of AtRBOHC/AtRBOHD. It is not known why eADP is ineffective. The pharmacological block that effectively eliminates eATP-activated Ca^2+^ influx across the plasma membrane only inhibits half of the intracellular ROS in roots caused by eATP [14]. The residual [Ca^2+^]_cyt_ elevation in *Atann1* roots in response to eATP could therefore have been sufficient to cause the observed normal level of ROS accumulation, particularly at the apex (Figure 6b). However, the results show that AtANN1 plays a role in limiting the spatial extent of the ROS increase, limiting its distal spread (Figure 5). AtANN1 can be a cytosolic protein in root cells [34] as well as being a plasma membrane protein. Recombinant AtANN1 has a very low level peroxidase activity in vitro [41], which suggests that loss of its activity would act to increase cytosolic ROS. However, this in vitro activity could have arisen from a co-purified protein [41]. Peroxide treatment of roots suppresses *AtANN1* expression [28], which is inconsistent with a role as a protective peroxidase. Annexin overexpression can protect against oxidative stress by elevating peroxidase, catalase and superoxide dismutase activities [42,43,44]. One possibility is that the AtANN1-dependent pathway spatially fine-tunes the expression or activity of one or more of the five peroxidases that are regulated by extracellular ATP [45]. Therefore, the *Atann1* mutant would exhibit the observed loss of spatial control of eATP-induced ROS accumulation.

Changes in gene expression were also examined to investigate the effect of *Atann1* in the downstream responses to eATP. eATP induction of *AtRBOHD* expression is *AtDORN1*-dependent in seedlings, suggesting [Ca^2+^]_cyt_ dependence [7], but here no effect of eATP on its expression was found in Col-0 or *Atann1* roots (Figure 7b). It could be that the effect of eATP treatment on *AtRBOHD* gene expression is more pronounced in leaves compared to the roots. Figure 7c reveals the potential importance of eATP signal regulation on ethylene production. AtACS6 is one of the many isoforms of 1-aminocyclopropane-1-carboxylix acid synthase (ACS) that is an important enzyme in the biosynthesis of ethylene. Its eATP-induced expression is *AtDORN1*-dependent [18]. Here, *AtACS6* expression was transiently upregulated in Col-0 whole roots. Transient up-regulation was also observed in previous studies over a similar time course [12,46]. The *Atann1* mutant failed to upregulate *AtACS6* expression compared to Col-0, indicating an important requirement for AtANN1 in this part of the eATP signalling pathway. A recent study showed that eATP-dependent ethylene production alleviates salinity stress by regulating Na^+^ and K^+^ homeostasis [47]. Salt stress itself promotes eATP accumulation by *Arabidopsis* roots [48] and *Atann1* is impaired in both the root’s transcriptional response to NaCl and the adaptive growth response [26,27]. It is possible, therefore, that a component of the salt stress response involves eATP’s acting through AtANN1 to up-regulate *ACS6* expression and ethylene production. Salt stress also up-regulates expression of *AtWRKY40* [49] and its up-regulation by eATP in roots is *AtDORN1*-dependent [7]. Both Col-0 and *Atann1* showed up-regulation of *AtWRKY40* expression by eATP, with *Atann1′*s exhibiting greater expression at 10 min than Col-0 (Figure 7d). This difference in the time course could relate more readily to the higher ROS in the mutant than the lesion in [Ca^2+^]_cyt_ response. With the demonstration that eATP-induced transcriptional responses can be affected by AtANN1, further studies should expand to the level of RNA_seq_ analysis to determine the extent to which this annexin is involved.

Although expressed in cotyledons and true leaves [32], no evidence was found here for the involvement of AtANN1 in the cotyledon eATP-induced [Ca^2+^]_cyt_ increase (Figure 8). For true leaves tested with eATP or eADP, only 1 mM eATP supported a significant difference between *Atann1* and Col-0 (Figure 9 and Figure 10). This is might be due to the temporal regulation of AtANN1 function and it seems that, in contrast to the root first [Ca^2+^]_cyt_ peak, AtANN1 is recruited to the pathway only at higher agonist concentration. It would be worthwhile to repeat these studies with another [Ca^2+^]_cyt_ reporter to afford spatial resolution and resolve any cell- or tissue-specific involvement of AtANN1.

**Figure 11:**
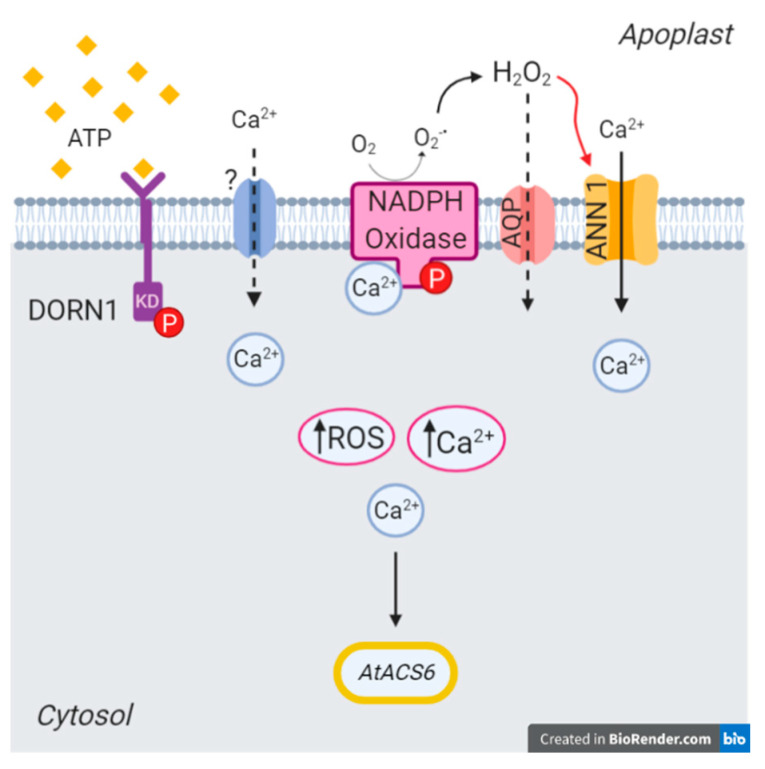
eATP perception by AtDORN1 at the root epidermal plasma membrane could be upstream of AtANN1 via production of extracellular ROS. Perception of eATP by AtDORN1 activates as yet unknown Ca^2+^ influx channels. AtDORN1 could phosphorylate an NADPH oxidase (AtRBOHC and AtRBOHD being the most likely candidates) with its kinase domain (KD) and the NADPH oxidase could also be activated by [Ca^2+^]_cyt_ at its EF hands. This would result in production of extracellular hydroxyl radicals that could readily be converted to peroxide (H_2_O_2_) or hydroxyl radicals [26,27]. These could promote AtANN1-mediated Ca^2+^ influx. Peroxide could enter the cytosol through aquaporins (AQP) [54], which could account for eATP-induced intracellular ROS accumulation. The latter is the clearest point for the divergence between eATP and eADP pathways. Decoding of [Ca^2+^]_cyt_ as a second messenger leads ultimately to a nuclear transcriptional response that can be impaired in *Atann1.* The position of AtP2K2 in such a pathway has yet to be tested. This original figure was created with BioRender.com.

AtANN1 is now known to be involved in [Ca^2+^]_cyt_ elevation in response to chitin, salinity stress, heat stress, extracellular hydroxyl radicals and H_2_O_2_ [26,27,28,50,51,52,53]. Here, in roots, it most likely operates downstream of AtDORN1, given the dependency of the root eATP- and eADP-induced [Ca^2+^]_cyt_ response on this receptor [5]. Studies on the root epidermal plasma membrane have indicated that AtANN1 can operate as an extracellular ROS-activated Ca^2+^-permeable channel and it is likely to act as such in salt stress signalling [26,27]. While AtANN1 function in eATP signalling may well differ from cell type to cell type, we propose that this is the most likely mode of action for eATP signalling in the root epidermis (Figure 11), especially given AtDORN1′s ability to activate the AtRBOHD plasma membrane NADPH oxidase that would generate extracellular ROS. This now requires testing. As eATP-and eADP-induced root [Ca^2+^]_cyt_ increase still occurred in *Atann1*, there remain Ca^2+^ channels downstream of AtDORN1 (and probably upstream of NADPH oxidases) to be discovered.

## 4. Materials and Methods

### 4.1. Plant Materials and Growth Conditions

The *annexin 1* loss of function mutant (*Atann1*) was in the Columbia (Col-0) wild type background with a T-DNA insertion in the third exon [26]. The *Atann1* line expressing the (apo)aequorin protein cytosolically under the 35S promoter was as described in [26]. Surface-sterilised seeds were sown on half-strength Murashige-Skoog (MS; Duchefa Biochemie, Haarlem, The Netherlands) medium (0.8% *w/v*) Bactoagar (BD Diagnostics VWR, Sparks, MD, USA); pH 5.6 with 0.1 M KOH +/− 1 mM ATP) in square petri dishes (12 cm × 12 cm, Greiner Bio-One, Frickenhausen, Germany) and incubated in the dark at 4 °C. After two days of stratification, plates were transferred into a growth chamber (PERCIVAL, CLF Plant Climatics, Emersacker, Germany) at 23 °C with a 16 h photoperiod (80 μmol m^−2^ s^−1^). Plants were grown vertically for excised root experiments or horizontally for leaf experiments.

### 4.2. Measurement of [Ca^2+^]_cyt_

*A. thaliana* (apo)aequorin-expressing samples were incubated in 100 μL of control solution (2 mM Bis-Tris Propane; 10 mM CaCl_2_; 0.1 mM KCl, pH 5.8 adjusted using 1 M Bis-Tris Propane and 0.5 M MES) containing 10 μL coelanterazine (10 μM; Nanolight Technology, Pinetop, AZ, USA) overnight in the dark at room temperature in a 96-well plate (Greiner Bio-One, Frickenhausen, Germany). All samples were washed with coelanterazine-free control solution before any luminescence measurement was taken and then placed (as individual samples) into a 96-well plate containing 100 μL fresh control solution. Luminescence (as an output of free cytosolic calcium ion, [Ca^2+^]_cyt_) upon different treatments was measured using a FLUOstar OPTIMA (BMG Labtech, Ortenberg, Germany) plate reader. All the treatment solutions were prepared as additions to the control solution and adjusted to pH 5.8 (1 M Bis-Tris Propane and 0.5 M MES) prior to measurement. After 35 s of initial background measurement, 100 μL of control or treatment solution was added and the luminescence measured every second for 155s. At the end of each measurement, 100 μL discharge solution (10% (*v/v*) ethanol; 1 M CaCl_2_) was added to quench the total (apo)aequorin luminescence in each sample. [Ca^2+^]_cyt_ was determined according to the calibration formula given in [55]. ATP (adenosine-5′-triphosphate, disodium salt trihydrate) and ADP (adenosine-5′-diphosphate, disodium salt dihydrate) were purchased from Melford Laboratories Ltd. (Ipswich, UK) whereas non-hydrolysable analogues ATPγS (adenosine 5′-[γ-thio] triphosphate tetralithium salt and ADPβS (Adenosine 5′-[β-thio] diphosphate trilithium salt) were purchased from Sigma-Aldrich (St. Louis, MO, USA).

### 4.3. Determination of Intracellular ROS

Accumulation of intracellular reactive oxygen species in individual roots was determined using 50 μM CM-H_2_DCFDA (5-(and-6-)-chloromethyl-2′,7′-dichlorodihydrofluorscein diacetate, acetyl ester; Molecular Probes/Invitrogen, Waltham, MA, USA), as described previously [14]. The assay medium comprised LSM medium [56]; when eATP was added, medium was supplemented with CaCl_2_ to counteract chelation. Images of roots were acquired with a Nikon SMZ 1500 microscope and a QImaging Retiga cooled 12-bit camera (www.qimaging.com).

### 4.4. Quantification of eATP-Induced Gene Expression

Seven-days-old Col-0 and *Atann1* were acclimatised for 1 h at room temperature in the light in 8 mL control solution (2 mM Bis-Tris Propane; 10 mM CaCl_2_; 0.1 mM KCl) pH 5.8 in separate small petri dishes (Thermo Scientific, Waltham, MA, USA). Either 8 mL of control solution or 2 mM ATP solution (prepared in control solution to give a final concentration of 1 mM) was added. After 10 or 30 min, seedlings were placed in 10 mL RNAlater solution (25 mM sodium citrate, 10 mM EDTA, 70 g ammonium sulphate/100 mL solution, pH 5.2). The root from each seedling was excised (in the RNAlater solution), dried briefly on filter paper and then frozen in liquid nitrogen. RNA extraction was carried out by using the RNEasy Plant Mini Kit (QIAGEN, Hilden, Germany) with a DNAse treatment additional step (RNAse free DNAse kit, QIAGEN, Hilden, Germany) and LiCl purification. Complementary DNA (cDNA) was synthesised by using the Quantitect Reverse Transcription kit (QIAGEN, Hilden, Germany) following the manufacturer’s protocol. Quantitative Polymerase Chain Reaction (qPCR) was done with a Rotor-Gene 3000 thermocycler with the Rotor-Gene^TM^ SYBR^®^ Green PCR Kit (QIAGEN, Hilden, Germany) according to the manufacturer’s protocol. cDNA final concentration was 5 ng with 0.25 μM final primer concentration. The qPCR programme was: Initial stage 95 °C for 5 min, 40 cycles of 95 °C for 5 s, 60 °C for 10 s. Melting curves were used to check for specific amplification (ramping from 55 °C to 95 °C with 1 °C rise each step and 5 s delay between steps). The primer pairs used in the qPCR reaction were as shown in Appendix A (Table A1). Raw data were analysed by using the ‘modlist’ and the ‘getPar’ function in R (qpcR package) [57]. Quantification (R value) was performed by using the average of the selected Ct and efficiency values using the formula E_sample_ = Efficiency ^(−Ct)^. Data were normalised with two different housekeeping genes *UBQ10* and *TUB4* using the formula R_sample_ = E_sample_/(sqrt(E_UBQ10 ×_ E_TUB4_)) [58].

### 4.5. Statistical Analysis

All the data collected were analysed with the R statistical programme (https://www.r-project.org). ANOVA, Student’s *t*-test or Welch two sample *t*-test were used for parametric tests whereas either Wilcoxon rank-sum test or Kruskal–Wallis test was used for non-parametric tests. Further comparison was analysed with Tukey’s HSD or Dunnett’s post-hoc test. A 95% confidence interval was used for all tests carried out.

## 5. Conclusions

This study has identified AtANN1 as a component in mediating the root increase of [Ca^2+^]_cyt_ in response to both eATP and eADP. It is postulated that AtANN1 might operate as an ROS-activated plasma membrane Ca^2+^ channel, downstream of AtDORN1. Since loss of *AtANN1* does not completely abolish the [Ca^2+^]_cyt_ increase, there are clearly other channels in the pathway. AtANN1 appears to regulate the spatial extent of eATP-induced intracellular ROS in the root. At the gene expression level, AtANN1 is involved in eATP-induced up-regulation of *AtACS6* and *AtWRKY40,* thus potentially directing eATP signalling towards ethylene production and salt stress tolerance.

## Data Availability

Materials are available on request to the corresponding author.

## Abbreviations

ACS	1-Aminocyclopropane-1-carboxylate synthase
ADPβS	Adenosine 5′-[β-thio] diphosphate
ANN1	Annexin1
ANOVA	Analysis of Variance
AQP	Aquaporins
ATPγS	Adenosine 5′-[γ-thio] triphosphate
AUC	Area Under the Curve
[Ca^2+^]_cyt_	Cytosolic free calcium ion
CAM	Calmodulin
CAMTA	Calmodulin-binding Transcription Activators
CAMV	Cauliflower Mosaic Virus
cDNA	Complementary DNA
CM-H_2_DCFDA	5-(and-6-)-Chloromethyl-2′,7′-dichlorodihydrofluorscein diacetate
DAMP	Damage Associated Molecular Pattern
DORN1	Does not Respond to Nucleotides1
eATP	Extracellular ATP
eADP	Extracellular ADP
H_2_O_2_	Hydrogen peroxide
MES	2-(N-morpholino) ethanesulfonic
MS	Murashige and Skoog nutrient medium
NADPH	Nicotinamide adenine dinucleotide phosphate
NO	Nitric oxide
PA	Phosphatidic Acid
qRT-PCR	Quantitative reverse transcription-polymerase chain reaction
RBOH	Respiratory burst homologue
ROS	Reactive oxygen species
RNAseq	RNA sequencing
SEM	Standard error of mean
T-DNA	Transfer DNA
Tris	Tris base, 2-amino-2-(hydroxymethyl)-1,3-propanediol
TUB4	Tubulin Beta Chain4
UBQ10	Polyubiquitin10
YC3.6	Yellow Cameleon 3.6

## Appendix A

**Table A1:** The forward and reverse sequences of primers used for the gene expression tests.

Gene	Forward (5′–3′)	Reverse (3′–5′)
*AtANN1* (AT1G35720.1)	TGTTCTTCGTTCAGCAATCAAC	GTACTCCTCTCCAATGACCTTC
*AtDORN1* (AT5G60300)	ATGGTCACATTGCCTGCAGAAG	TCCCTCTTTACAGGCTGGACTCTC
*AtRBOHD* (AT5G47910)	ATGATCAAGGTGGCTGTTTACCC	ATCCTTGTGGCTTCGTCATGTG
*AtACS6* (AT4G11280)	TATCCAGGGTTTGATAGAGA	TCCACCGTAATCTTGAACC
*AtWRKY40* (AT1G80840)	AGCTTCTGACACTACCCTCGTTG	TTGACAGAACAGCTTGGAGCAC
*AtUBQ10* (AT4G05320)	CCGACTACAACATTCAGAAGGA	TCAGAACTCTCCACCTCCAAA
*AtTUB4* (AT5G44340)	AGGGAAACGAAGACAGCAAG	GCTCGCTAATCCTACCTTTGG
